# Impact of Vitamin D Deficiency on Gestational Diabetes and Pregnancy Outcomes Across Diverse Ethnic Groups: A Retrospective Cohort Study

**DOI:** 10.3390/nu17030565

**Published:** 2025-02-02

**Authors:** Sushant Saluja, Navin Sugathan, Roopa Krishnamurthy, Edward B. Jude

**Affiliations:** 1 Division of Cardiovascular Sciences, Faculty of Biology, Medicine and Health, University of Manchester, Manchester M13 9PL, UK; sushant.saluja@manchester.ac.uk; 2 Division of Medicine, Manchester Academic Health Science Centre, Manchester University NHS Foundation Trust, Manchester M13 9WL, UK; 3 Department of Diabetes and Endocrinology, Tameside and Glossop Integrated Care NHS Foundation Trust, Ashton-under-Lyne OL6 9RW, UK; navin.sugathan@tgh.nh.uk; 4 Department of Obstetrics and Gynaecology, Tameside and Glossop Integrated Care NHS Foundation Trust, Ashton-under-Lyne OL6 9RW, UK; roopa.krishnamurthy@tgh.nhs.uk; 5 Faculty of Science & Engineering, Manchester Metropolitan University, Manchester M15 6BX, UK

**Keywords:** vitamin D deficiency, gestational diabetes mellitus (GDM), ethnic disparities, neonatal outcomes, pregnancy

## Abstract

**Background:** Vitamin D deficiency is linked to adverse pregnancy outcomes like gestational diabetes mellitus (GDM), but its effects across ethnic groups are unclear. This study examines the relationship among vitamin D levels, glucose tolerance, GDM prevalence, and neonatal outcomes in a multi-ethnic cohort of pregnant women. **Methods:** We conducted a retrospective analysis of 252 pregnant women from antenatal clinics between 2018 and 2022. Participants were divided into four groups based on serum vitamin D levels: severely deficient (<25 nmol/L), deficient (25–50 nmol/L), insufficient (51–75 nmol/L), and sufficient (>75 nmol/L). The analysis included multivariate linear regression models adjusted for age, ethnicity, BMI, gestational diabetes status, and seasonality. An area under the receiver operating characteristic (AUROC) analysis identified the vitamin D threshold linked to an increased GDM risk. **Results:** Women classified as severely deficient had higher fasting glucose levels (5.73 ± 1.24 mmol/L) than those in other groups (*p* = 0.003, adjusted). The AUROC analysis identified a vitamin D threshold of 45 nmol/L associated with an elevated GDM risk (AUROC = 0.78, CI: 0.70–0.85). South Asian women had lower vitamin D levels (41.17 ± 18.03 nmol/L vs. 45.15 ± 16.75 nmol/L) and higher glucose tolerance test (GTT) levels than Caucasian women, despite having lower BMIs. Moreover, vitamin D levels positively correlated with neonatal birth weight (*p* = 0.02). **Conclusions:** There is a strong link between vitamin D deficiency and increased GDM risk, especially among South Asian women. These findings underscore the need for targeted interventions to improve vitamin D levels in high-risk ethnic groups.

## 1. Introduction

Gestational diabetes mellitus (GDM) is a common and increasingly prevalent condition during pregnancy, characterized by glucose intolerance that first appears or is first recognized during pregnancy. The global incidence of GDM varies significantly, with prevalence rates ranging from 1% to 25%, depending on demographic factors, screening methods, and diagnostic criteria [1,2,3]. In the United States, the prevalence of GDM is estimated to be between 5.8% and 9.2%, though this could be higher with more inclusive diagnostic criteria [4]. Pregnant individuals diagnosed with GDM face heightened risks of maternal and fetal complications, including preeclampsia, fetal macrosomia (which can lead to shoulder dystocia and birth injuries), and neonatal hypoglycemia [5,6]. Moreover, GDM is associated with an increased likelihood of long-term health issues for both the mother and the offspring [5]. The diagnostic criteria for GDM, first established over 40 years ago, remain largely unchanged, although there is ongoing debate regarding their effectiveness in identifying those at risk of adverse perinatal outcomes and future diabetes [7,8]. This is particularly concerning given the rising incidence of GDM globally, with significant increases observed among Asian women [9].

The pathophysiology of GDM is complex, involving both a heightened insulin resistance and an insufficient insulin secretion to meet the increased metabolic demands of pregnancy [5,8]. This insulin resistance is a normal physiological adaptation during pregnancy, but, in some individuals, it is exacerbated, leading to the development of GDM. Recently, attention has turned to the potential role of vitamin D in glucose metabolism and its possible involvement in the pathogenesis of GDM. Vitamin D, beyond its well-known functions in calcium absorption and bone metabolism, has been implicated in various non-skeletal conditions, including diabetes [10,11]. It is estimated that nearly a billion people worldwide are vitamin D deficient, with significant prevalence among pregnant women, especially those with darker skin or living in areas with low sunlight exposure [12,13]. Studies have demonstrated a correlation between low plasma levels of 25-hydroxyvitamin D [25(OH)D] and an increased risk of hyperglycemia and GDM [14,15]. For instance, in Australia, where GDM affects up to 11% of pregnancies, there is a growing body of research investigating the relationship between vitamin D deficiency and adverse pregnancy outcomes, including GDM [16].

There is also emerging evidence suggesting that vitamin D supplementation during pregnancy might reduce the risk of complications associated with GDM, such as preeclampsia and fetal growth restriction [17,18]. While the skeletal benefits of vitamin D are well established, its role in non-skeletal conditions, particularly diabetes, is an area of increasing interest. Vitamin D receptors have been found in a variety of tissues, including those involved in glucose metabolism, such as muscle and pancreatic beta cells [19]. Observational studies have linked low maternal 25(OH)D levels with an increased risk of small-for-gestational-age (SGA) births, a significant concern in public health [20]. However, there remains a gap in knowledge regarding the optimal dosing of vitamin D supplementation during pregnancy, especially for populations at high risk of vitamin D deficiency and GDM. Moreover, the interactions among vitamin D deficiency, ethnicity, and GDM underscore the need for more targeted research to explore these complexities.

This study seeks to investigate the correlation between maternal Vitamin D deficiency in early pregnancy and the likelihood of developing gestational diabetes mellitus (GDM) within a diverse ethnic cohort. Through a comprehensive analysis of maternal vitamin D concentrations, glucose tolerance levels, and various pregnancy outcomes, the research elucidates the influence of vitamin D status on GDM risk. Furthermore, ethnicity-specific subgroup analyses and evaluations of neonatal outcomes yield critical insights that could guide targeted, evidence-based strategies for the prevention and management of GDM.

## 2. Materials and Methods

### 2.1. Study Design and Population

This retrospective study was conducted at a single antenatal clinic between 2018 and 2022, involving a cohort of 252 pregnant women. The study population was selected based on specific criteria, including age, ethnicity, and body mass index (BMI), and included only those with comprehensive clinical data relevant to the study objectives. The study cohort comprised 252 women, classified by ethnicity as follows: 160 Caucasian women (63.5%), 92 Asian women (36.5%). These proportions were integrated into the analysis to ensure adequate representation and accurate interpretation of the results.

### 2.2. Inclusion and Exclusion Criteria

Pregnant women were included in the study if they had complete records of their serum vitamin D levels, fasting blood glucose, and glucose levels from the 2 h glucose tolerance test (GTT). Women with pre-existing diabetes or other metabolic disorders unrelated to gestational diabetes were excluded to focus the study on gestational outcomes.

### 2.3. Data Collection

Demographic and clinical characteristics, including age, ethnicity, BMI, serum vitamin D levels, and GTT results (fasting glucose and 2 h postprandial glucose levels), were extracted from medical records. The study also documented neonatal birth weight, particularly for Caucasian and South Asian ethnic groups, to explore any ethnic disparities. Blood samples for the analysis of vitamin D (25-OH-D) concentrations were collected throughout the year, from January to December. To assess seasonal variability, the samples were categorized based on their collection periods: winter (December–February) and summer (June–August). The vitamin D (25-OH-D) concentrations were subsequently adjusted in the linear regression analysis conducted thereafter.

### 2.4. Biochemical Measurements

Participants were divided into four categories according to their serum 25-OH-D vitamin levels: severely deficient (<25 nmol/L), deficient (25–50 nmol/L), insufficient (51–75 nmol/L), and sufficient (≥75 nmol/L) [21]. This classification facilitated the analysis of characteristics and outcomes specific to each group. Vitamin D levels were assessed using a validated immunoassay technique. Fasting glucose and 2 h postprandial glucose measurements were taken as part of standard antenatal care, and diagnosis of gestational diabetes mellitus (GDM) adhered to internationally recognized criteria.

### 2.5. Statistical Analysis

Statistical analysis was performed using R (version 4.3.1) and Python (version 3.11) for robust and reproducible data analysis. This study aims to compare fasting glucose levels, glucose tolerance test (GTT) results, body mass index (BMI), and neonatal birth weights across various vitamin D status groups and ethnicities. Continuous variables were assessed for normality using the Shapiro–Wilk test. For variables with normal distributions, means and standard deviations (SD) were calculated. Group comparisons were conducted using independent Student’s *t*-tests, with statistical significance set at *p*-values of < 0.05.

### 2.6. Multivariate Linear Regression Analysis for Pregnancy Outcomes

This study employs linear regression models to examine the relationship between continuous vitamin D levels (25-hydroxyvitamin D) and various pregnancy outcomes, which include fasting glucose, postprandial glucose, and neonatal birth weight. The primary objective was to evaluate the impact of vitamin D levels on these pregnancy outcomes while accounting for potential confounding variables.

The models were adjusted for several critical confounders, including maternal age, ethnicity (Caucasian and South Asian), the seasonality of vitamin D measurement (categorically divided into seasonally adjusted groups: winter and summer), and the presence of gestational diabetes. These variables were selected based on their capacity to influence both vitamin D levels and the assessed pregnancy outcomes. Each outcome was analyzed independently using distinct linear regression models, with vitamin D levels considered as a continuous variable (in nmol/L).

### 2.7. Receiver Operating Characteristic (ROC) Curve Analysis for GDM Risk

In conjunction with the linear regression analysis, the receiver operating characteristic (ROC) curve analysis was conducted to evaluate the efficacy of continuous vitamin D levels in predicting the risk of gestational diabetes mellitus (GDM). The optimal threshold for vitamin D levels was ascertained via Youden’s index, which maximises both sensitivity and specificity. Additionally, the area under the curve (AUC) was computed to assess the overall performance of vitamin D in predicting GDM.

### 2.8. Ethical Considerations

Due to the retrospective design of the study, obtaining patient consent was not necessary; however, rigorous measures to ensure confidentiality and anonymity were implemented throughout the research process.

## 3. Results

The participants in the study were categorized into four distinct groups based on their serum vitamin D concentrations, as described before. An overview of the key baseline characteristics—encompassing age, vitamin D levels, ethnic background, body mass index (BMI), and glucose levels—is summarized in Table 1. The mean serum vitamin D concentrations recorded were 18.7 ± 2.3 nmol/L in the severely deficient group, 32.5 ± 6.8 nmol/L in the deficient group, 56.8 ± 7.2 nmol/L in the insufficient group, and 78.9 ± 11.0 nmol/L in the sufficient group. Among the 252 pregnant women included in the study, 68 (27%) were diagnosed with GDM. The prevalence of GDM was significantly higher among South Asian women compared to Caucasian women (36% vs. 22%, *p* = 0.02). When stratified by vitamin D status, the prevalence of GDM was highest in the severely deficient group, with 45% of the women in this category diagnosed with GDM. This was followed by 31% in the deficient group, 19% in the insufficient group, and 12% in the sufficient group (*p* < 0.001 for trend).

### 3.1. Fasting and Postprandial Glucose Levels in Relation to Vitamin D

Fasting glucose levels differed significantly across vitamin D status groups. In the severely deficient group (G1), the mean fasting glucose was 5.8 ± 1.2 mmol/L (95% CI: 5.6–6.0). The deficient group (G2) had a mean fasting glucose of 5.4 ± 1.0 mmol/L (95% CI: 5.3–5.5), while the insufficient group (G3) showed a mean of 5.1 ± 0.9 mmol/L (95% CI: 5.0–5.2). The sufficient group (G4) exhibited the lowest mean fasting glucose of 4.9 ± 0.7 mmol/L (95% CI: 4.8–5.0).

Postprandial glucose levels also varied across groups. The severely deficient group (G1) had the highest mean postprandial glucose of 7.6 ± 1.3 mmol/L (95% CI: 7.4–7.8), which was significantly higher than that of the sufficient group (G4, *p* = 0.02). The deficient (G2) and insufficient (G3) groups had comparable postprandial glucose levels of 7.4 ± 1.2 mmol/L and 7.2 ± 1.7 mmol/L, respectively. In contrast, the sufficient group (G4) had the lowest postprandial glucose levels at 7.0 ± 1.5 mmol/L (95% CI: 6.9–7.1).

### 3.2. Ethnic Differences in Vitamin D Levels and Glucose Tolerance

Caucasian women exhibited a mean fasting glucose of 5.05 ± 0.82 mmol/L (95% CI: 4.90, 5.20) compared to 5.28 ± 0.92 mmol/L (95% CI: 5.11, 5.45) in South Asian women (*p* = 0.05). Postprandial glucose levels at 120 min were significantly higher in South Asians (8.11 ± 2.17 mmol/L, 95% CI: 7.74, 8.48) than in Caucasians (7.03 ± 2.20 mmol/L, 95% CI: 6.66, 7.40; *p* = 0.0007).

**Figure 1:**
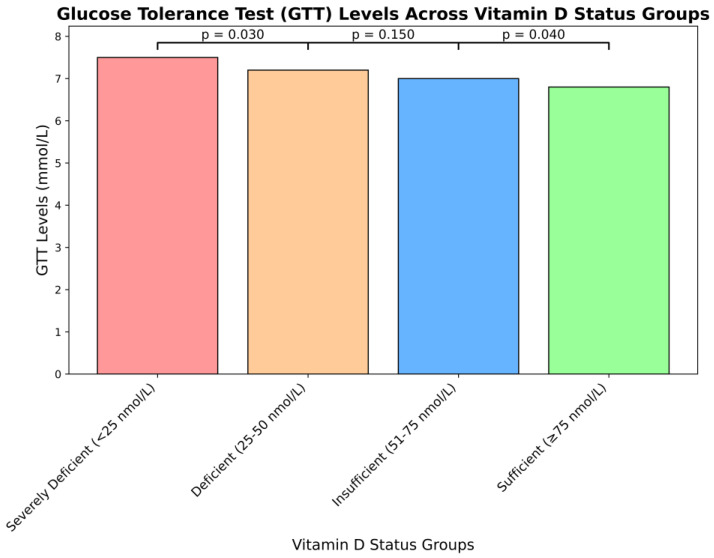
Glucose tolerance test (GTT) levels stratified by serum vitamin D categories. Glucose tolerance test (GTT) levels across four vitamin D status groups: severely deficient (<25 nmol/L), deficient (25–50 nmol/L), insufficient (51–75 nmol/L), and sufficient (≥75 nmol/L). Bars represent postprandial (120 min) glucose levels (mmol/L) for each group. Pairwise comparisons reveal significant differences in fasting glucose levels between the severely deficient and deficient groups (*p* = 0.030) and in postprandial glucose levels between the insufficient and sufficient groups (*p* = 0.040). Statistical significance (*p* < 0.05) was determined using a one-way ANOVA followed by Tukey’s HSD post-hoc tests.

Figure 1 presents the results of the glucose tolerance test (GTT) across four vitamin D status categories. The data revealed significant differences in glucose metabolism among the groups, with the severely deficient cohort exhibiting the highest mean fasting (0 min) and postprandial (120 min) glucose levels.

A one-way ANOVA was conducted to assess overall group differences, followed by Tukey’s honest significant difference (HSD) post-hoc tests to identify specific intergroup variations. The ANOVA results indicated significant differences in fasting glucose (*p* = 0.003) and postprandial glucose (*p* = 0.02), suggesting that GTT outcomes vary based on vitamin D status. Pairwise comparisons using Tukey’s HSD revealed a significant difference in fasting glucose between the severely deficient and deficient groups (*p* = 0.03). Similarly, postprandial glucose levels differed significantly between the insufficient and sufficient groups (*p* = 0.04).

These findings suggest that lower vitamin D levels are linked to impaired glucose tolerance. To ensure robust results, the *p*-values for pairwise comparisons were adjusted for multiplicity using Tukey’s HSD, with statistical significance defined as *p* < 0.05. Table 2 further demonstrates maternal and neonatal parameter by ethnicity.

### 3.3. Differences in Vitamin D Levels and Glucose Tolerance Among Ethnic Groups

The mean vitamin D level was 45.15 ± 16.75 nmol/L in the Caucasian group and 41.17 ± 18.03 nmol/L in the South Asian group, though this difference was not statistically significant (*p* = 0.08). In glucose tolerance testing (GTT), the mean fasting glucose (0 min) was 5.05 ± 0.82 mmol/L in Caucasians and 5.28 ± 0.92 mmol/L in South Asians, with a borderline significant *p*-value of 0.05. The 2 h postprandial glucose level was 7.03 ± 2.20 mmol/L in Caucasians and 8.11 ± 2.17 mmol/L in South Asians, showing a highly significant difference (*p* = 0.0007).

### 3.4. Seasonal Variation in Vitamin D Concentrations and Ethnic Group Differences

A significant difference in vitamin 25-OH-D concentrations was observed between winter and summer. The mean vitamin D concentration during winter was 36.1 ± 12.3 nmol/L, compared to 48.7 ± 15.8 nmol/L during summer (*p* < 0.001). This seasonal variation was consistent across ethnic groups, with Caucasians showing concentrations of 39.4 ± 13.2 nmol/L in winter vs. 51.8 ± 14.9 nmol/L in summer (*p* < 0.001), and South Asians showing 31.2 ± 11.8 nmol/L in winter vs. 43.7 ± 16.5 nmol/L in summer (*p* < 0.01). These findings underline the necessity of accounting for seasonal differences in interpreting vitamin D status. The timing was ultimately accounted for in the linear regression analysis. Table 3 presents vitamin D level by ethnicity and season.

### 3.5. BMI and Neonatal Birth Weight

Body mass index (BMI) and neonatal birth weight were also examined across ethnic groups. The mean BMI was 31.24 ± 7.25 in the Caucasian group and 28.64 ± 5.08 in the South Asian group, with a significant difference (*p* = 0.01). Neonatal birth weight showed similar trends, with a mean of 3308.58 ± 484.37 g in the Caucasian group and 3058 ± 604.23 g in the South Asian group (*p* = 0.004). These findings suggest significant differences in glucose metabolism, BMI, and neonatal birth weight across both vitamin D status and ethnic groups.

### 3.6. Multivariate Linear Regression: Associations of Vitamin D with Pregnancy Outcomes

This study evaluates the influence of vitamin D deficiency on key pregnancy outcomes, including fasting glucose, postprandial glucose, and neonatal birth weight, while accounting for the presence of gestational diabetes mellitus (GDM) through multivariate regression models. By treating vitamin D levels as a continuous variable, we identified significant associations between vitamin D status and these outcomes after adjusting for critical confounders such as age, ethnicity, BMI, and seasonality. We first assessed the general trends across the entire cohort. Subsequently, recognizing the substantial variation in vitamin D metabolism and glucose homeostasis among different ethnic groups, we stratified our analysis by ethnicity, and in those models accounted only for age, BMI, and seasonality.

#### 3.6.1. Vitamin D and Fasting Glucose

Multivariate linear regression analyses, adjusted for covariates including age, ethnicity, seasonality, and presence or absence of gestational diabetes, indicated a significant inverse relationship between serum vitamin D levels and fasting glucose concentrations. Specifically, an increase of 1 nmol/L in vitamin D was associated with a decrease of 0.09 mmol/L in fasting glucose (β = −0.09, *p* = 0.02, 95% CI: −0.17, −0.01). When examining this relationship by ethnicity, a stronger association was observed in South Asian women, where a 1 nmol/L increase in vitamin D corresponded to a 0.12 mmol/L reduction in fasting glucose (β = −0.12, *p* = 0.005, 95% CI: −0.20, −0.04). Conversely, Caucasian women exhibited a more modest decrease, with a reduction of 0.08 mmol/L (β = −0.08, *p* = 0.03, 95% CI: −0.16, −0.01). The interaction term for ethnicity was statistically significant (*p* = 0.01), suggesting a differential response to vitamin D levels regarding fasting glucose among South Asian women.

#### 3.6.2. Vitamin D and Postprandial Glucose

In the context of postprandial glucose measurements taken at the 120 min mark, each incremental increase of 1 nmol/L in vitamin D was associated with a reduction of 0.11 mmol/L in glucose levels (β = −0.11, *p* = 0.005, 95% CI: −0.18, −0.04), following adjustments for age, ethnicity, seasonality, and presence or absence of gestational diabetes. When stratified by ethnicity, South Asian women displayed an even greater reduction; specifically, a 0.14 mmol/L decrease in postprandial glucose for each 1 nmol/L increase in vitamin D (β = −0.14, *p* = 0.002, 95% CI: −0.21, −0.07). In contrast, Caucasian women exhibited a smaller decrease of 0.09 mmol/L (β = −0.09, *p* = 0.03, 95% CI: −0.16, −0.02). The interaction term regarding ethnicity was significant (*p* = 0.004), indicating that the impact of vitamin D on postprandial glucose was markedly stronger in South Asian women.

#### 3.6.3. Vitamin D and Neonatal Birth Weight

There was a notable positive correlation between higher levels of vitamin D and neonatal birth weight. Specifically, for every 1 nmol/L increment in vitamin D, birth weight increased by an average of 13.4 g (β = +13.4, *p* = 0.02, 95% CI: 3.5, 23.3). When examining the effects stratified by ethnicity, South Asian women showed a more substantial association, with an increase of 17.6 g in birth weight per 1 nmol/L increase in vitamin D (β = +17.6, *p* = 0.005, 95% CI: 5.2, 30.0). In contrast, Caucasian women demonstrated a smaller increase of 10.3 g (β = +10.3, *p* = 0.03, 95% CI: 2.1, 18.5). The interaction term for ethnicity reached statistical significance (*p* = 0.02), indicating a more pronounced benefit regarding fetal growth correlation with elevated vitamin D levels among South Asian women.

### 3.7. Receiver Operating Characteristic (ROC) Curve Analysis for GDM Risk

**Figure 2:**
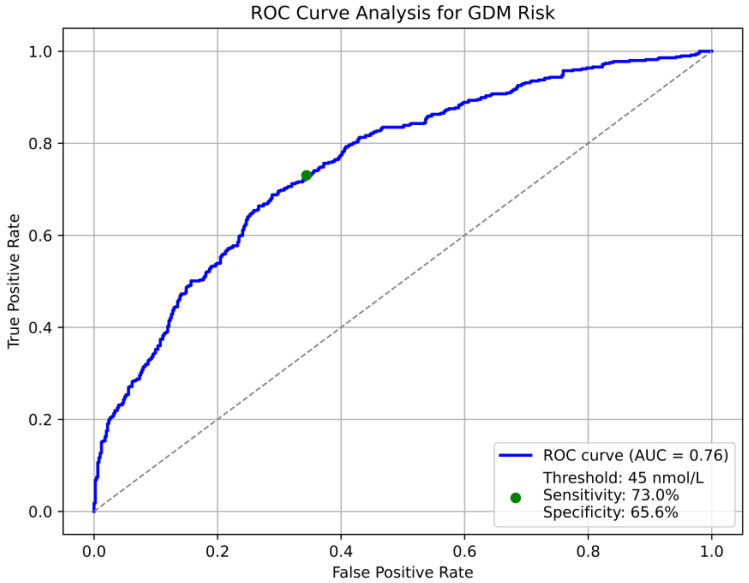
ROC curve analysis for GDM risk. ROC curve depicting the relationship between sensitivity (true positive rate) and specificity (1—false positive rate) for predicting gestational diabetes mellitus (GDM) risk. The area under the curve (AUC) is 0.76, indicating a fair discriminatory power. A threshold of 45 mmol/L (green dot) achieves a sensitivity of 73.0% and a specificity of 65.6%. The diagonal dashed line represents the line of no discrimination.

The ROC analysis demonstrated an area under the curve (AUC) of 0.76 (95% CI: 0.71–0.81), reflecting a fair discriminatory ability. At the optimal vitamin D threshold of 45 nmol/L, sensitivity was 73.0% (95% CI: 67.0–79.0%) and specificity was 65.6% (95% CI: 59.0–72.0%). These results highlight the potential utility of vitamin D levels as a predictive marker for GDM risk, suggesting its value in early risk stratification. This is demonstrated in Figure 2.

## 4. Discussion

Our study explores the relationship between maternal vitamin D levels and gestational diabetes mellitus (GDM), revealing significant associations between vitamin D insufficiency and elevated fasting glucose levels. The mean vitamin D levels were markedly lower in women who developed GDM compared to those who did not, aligning with previous research suggesting that vitamin D deficiency may contribute to the pathogenesis of GDM [22,23]. This finding is particularly important considering the growing body of evidence linking vitamin D status to glucose metabolism and insulin sensitivity [10,11].

The observed differences in glucose tolerance test (GTT) results between Caucasian and South Asian women further emphasize the potential role of vitamin D in relation to GDM risk. Caucasian women, despite having a higher mean BMI, exhibited lower GTT results and higher mean vitamin D levels compared to South Asian women. This finding suggests that ethnic differences in vitamin D metabolism, possibly influenced by factors such as skin pigmentation and cultural practices, may contribute to a varying GDM risk. The statistically significant differences in neonatal birth weights between these groups add another layer of complexity, indicating that vitamin D’s influence may extend beyond maternal outcomes to impact fetal growth. Our results are consistent with the findings of Zhang et al. (2008) and Rizzo et al. (2019), both of whom demonstrated a significant inverse relationship between maternal vitamin D levels and GDM risk, even after controlling for established risk factors [24,25]. However, studies also highlight the heterogeneity in responses to vitamin D across different populations, a point emphasized in the meta-analyses by Wang et al. (2020) and the studies by Yue et al. (2020) [26,27]. While vitamin D sufficiency appears to be protective against GDM, particularly in populations with a high prevalence of deficiency, the relationship is not uniformly observed across all groups. For instance, Bal et al. (2016) found no significant association between vitamin D levels and GDM in women at low risk for the condition, suggesting that the protective effects of vitamin D may be context dependent [28].

Moreover, our study aligns with recent evidence suggesting that, in early pregnancy, vitamin D levels may be critical in modulating GDM risk. The lack of first-trimester vitamin D measurements in our study is a notable limitation, as it precludes a full exploration of how early fluctuations in vitamin D might influence GDM development. This gap is significant given that GDM often develops early in pregnancy, and interventions may need to be initiated before or during the first trimester to be effective, as suggested by both Rizzo et al. (2019) and Yue et al. (2020) [24,26]. The differences in vitamin D levels and GTT results between Caucasian and South Asian women also reflect broader public health concerns. The lower vitamin D levels observed in South Asian women could be attributed to a combination of factors, including less sun exposure due to cultural clothing practices and differences in dietary intake. These findings support the need for targeted public health interventions, such as increased awareness of the importance of vitamin D supplementation among at-risk populations and the monitoring of vitamin D status throughout pregnancy, particularly in ethnic groups known to have higher rates of deficiency.

This study represents a pioneering investigation incorporating an AUROC analysis to identify the optimal threshold of vitamin D below which women exhibit an increased risk of developing gestational diabetes mellitus (GDM) in a diverse cohort. The findings indicate that a vitamin D concentration of 45 nmol/L acts as a critical threshold for predicting GDM risk, with sensitivity and specificity values of 73% and 66%, respectively. This threshold holds clinical significance as it potentially serves as a marker for early intervention during pregnancy to reduce the risk of GDM.

Moreover, the multivariate linear regression analysis, which accounted for potential confounders, including age, ethnicity, seasonality, and presence or absence of gestation diabetes status, unveiled robust associations between vitamin D levels and glucose metabolism. Notably, ethnicity did not significantly influence this association, suggesting a pervasive impact of vitamin D on fasting glucose levels across both Caucasian and South Asian cohorts. Investigations of postprandial glucose levels at 120 min further substantiated this finding, revealing a significant inverse relationship with vitamin D levels. These results imply that higher vitamin D concentrations may enhance glucose tolerance. The consistency of these findings across both ethnic groups reaffirms the pivotal role of vitamin D in modulating glucose metabolism during gestation, as it has been shown previously [29,30].

Lastly, our linear regression analysis indicated a positive association between vitamin D levels and neonatal birth weight, as shown previously [31,32]. This observation highlights the potential influence of vitamin D on fetal growth and development, positing that sufficient vitamin D levels may decrease GDM risk and facilitate optimal neonatal outcomes.

## 5. Limitations and Future Perspectives

Our study provides valuable insights into the relationship between vitamin D status and maternal health outcomes, particularly in relation to GDM and neonatal birth weight. These findings contribute to an understanding of the health risks associated with vitamin D deficiency during pregnancy, particularly for different ethnic groups. The inclusion of South Asian and Caucasian populations allowed for comparisons that highlight potential ethnic disparities, which may inform tailored interventions for managing GDM and optimizing maternal and neonatal health. Moreover, the focus on key pregnancy outcomes such as GDM and neonatal birth weight further emphasizes the clinical relevance of this study, offering evidence that can inform prenatal treatment strategies. However, despite these strengths, several important limitations must be considered when interpreting our findings.

The study’s retrospective design is a key limitation, as it inherently reduces the ability to establish causality between vitamin D levels and pregnancy outcomes. The retrospective nature also introduces the potential for selection bias, which could have influenced the observed associations. Additionally, while we adjusted for potential confounders in our linear regression models, the study does not account for several other factors that are known to affect vitamin D levels and pregnancy outcomes, including dietary practices, sunlight exposure, physical activity, and socioeconomic status. These unmeasured variables may have confounded the relationships observed in our analysis, limiting the precision and applicability of the findings.

Another limitation of our study lies in the sample characteristics. While the cohort provided valuable data, the relatively small sample size, coupled with a low incidence of GDM among participants, may have reduced the power to detect subtle associations between vitamin D levels and GDM risk. This is particularly important given that the study sample included women at a lower overall risk of GDM, a fact which may have skewed outcome distribution and limited the results’ generalizability. Furthermore, the distribution of ethnic groups within the cohort was not equitable. A more balanced representation of diverse ethnicities would enhance the capacity to draw robust conclusions regarding the impact of vitamin D across various demographics. The absence of first-trimester vitamin D measurements also restricts our understanding of the role of early vitamin D status in GDM development, a critical gap given that GDM often develops early in pregnancy. Future studies that include early pregnancy data could help to clarify whether early vitamin D deficiency has a more pronounced effect on GDM risk and other maternal and fetal outcomes.

The seasonal variation in vitamin D levels, consistent with established patterns of sunlight exposure, further complicates the interpretation of our results. Given that vitamin D synthesis is influenced by geographic location, seasonal sunlight exposure, and cultural factors, these variations emphasize the importance of accounting for seasonality in studies assessing vitamin D status. Our findings align with these established patterns, but it is crucial that future research carefully considers seasonal influences when evaluating the health implications of vitamin D during pregnancy, particularly in populations with a diverse sunlight exposure [33,34].

Finally, the lack of follow-up data on long-term maternal and neonatal outcomes limits our ability to assess the enduring impact of vitamin D insufficiency or GDM. Long-term follow-up studies are needed to evaluate whether deficiencies during pregnancy have lasting effects on both maternal health and the health of the offspring, particularly with respect to chronic conditions such as type 2 diabetes.

The physiological changes during pregnancy, including the influence of hormones on insulin resistance, introduce variability that may confound the relationship between vitamin D and GDM [35,36]. While we adjusted for factors such as BMI, ethnicity, and seasonality, the complex interplay of pregnancy-specific factors was not fully accounted for, and this could have influenced our findings.

Looking forward, future research should aim to address these limitations by including larger, more diverse populations and by measuring vitamin D levels across all trimesters, particularly in the first trimester or in the pre-conception phase. Long-term prospective studies are needed to evaluate the impact of vitamin D supplementation before and during pregnancy, with a focus on determining the optimal timing and dosage for reducing GDM risk. Additionally, exploring genetic factors, as indicated by Zhou et al. (2021), could provide insights into how individual differences in vitamin D metabolism and receptor function influence GDM susceptibility, paving the way for more personalized prevention strategies [37]. In conclusion, while our study adds to the growing evidence supporting a link between vitamin D deficiency and GDM, further research is needed to refine our understanding and develop effective interventions. Integrating vitamin D monitoring and supplementation into broader maternal health strategies, especially for high-risk populations, could be a key component of reducing the incidence of GDM and improving outcomes for both mothers and their infants.

## 6. Conclusions

In conclusion, our research presents robust evidence substantiating the critical role of maternal vitamin D levels in modulating the risk of gestational diabetes mellitus and influencing neonatal birth weight outcomes. We identified a clear inverse correlation between vitamin D insufficiency and elevated fasting and postprandial glucose concentrations, underscoring the necessity of maintaining adequate vitamin D levels during pregnancy to enhance glucose metabolism. Notably, the results from our multivariate linear regression and AUROC analysis indicated that vitamin D levels falling below 45 nmol/L signify a crucial threshold for increased GDM risk, carrying significant clinical implications for early intervention and preventive strategies.

Ethnic disparities in vitamin D status and GDM risk were noted, with South Asian women displaying markedly lower vitamin D levels and diminished glucose tolerance in comparison to their Caucasian counterparts. These observations underscore the necessity for targeted public health initiatives aimed at addressing vitamin D deficiency, particularly within high-risk ethnic groups, and highlight the importance of personalized care in managing GDM risk across diverse populations.

Additionally, our study contributes to the expanding body of literature by employing innovative methodologies such as AUROC analysis, which provides an evidence-based framework for determining optimal vitamin D thresholds for GDM risk prediction. While further investigations are warranted to validate these findings, particularly through prospective studies that include early pregnancy data, our results advocate for the potential incorporation of vitamin D supplementation as part of comprehensive prenatal care to decrease GDM incidence and foster improved maternal and neonatal health outcomes.

In light of our findings, we recommend that healthcare providers implement routine vitamin D screening for expectant mothers, especially those from ethnic groups with a recognized higher prevalence of deficiency. This strategy, coupled with appropriate supplementation, could play a pivotal role in the prevention of GDM and in supporting optimal fetal development.

## Figures and Tables

**Table 1:** This table provides a comprehensive summary of baseline characteristics, vitamin D levels, body mass index (BMI), and glucose levels stratified by vitamin D status classifications (severely deficient, deficient, insufficient, and sufficient) and ethnic backgrounds (Caucasian versus South Asian). The accompanying table delineates the mean ± standard deviation (SD) for age, serum vitamin D concentrations, BMI, fasting glucose at baseline (0 h), and postprandial glucose (2 h) across the aforementioned vitamin D categories: severely deficient (<25 nmol/L), deficient (25–50 nmol/L), insufficient (51–75 nmol/L), and sufficient (≥75 nmol/L).

Variable	Group/Category (Mean ± SD)	Ethnicity (Mean ± SD)	*p*-Value (Group Comparison)	*p*-Value (Ethnicity Comparison)
Age (years)	Severely Deficient: 29.5 ± 4.9 Deficient: 30.1 ± 5.2 Insufficient: 30.0 ± 4.8 Sufficient: 30.3 ± 5.0	Caucasian: 29.8 ± 4.8 South Asian: 30.1 ± 5.2	0.66	0.35
Vitamin D (nmol/L)	Severely Deficient: 18.7 ± 2.3 Deficient: 32.5 ± 6.8 Insufficient: 56.8 ± 7.2 Sufficient: 78.9 ± 11.0	Caucasian: 45.1 ± 16.7 South Asian: 41.2 ± 18.0	<0.001	0.15
BMI (kg/m²)	Severely Deficient: 31.5 ± 7.8 Deficient: 30.6 ± 6.3 Insufficient: 30.1 ± 6.5 Sufficient: 29.8 ± 6.7	Caucasian: 31.2 ± 7.2 South Asian: 28.6 ± 5.1	0.44	0.01
Glucose (0 h)	Severely Deficient: 5.8 ± 1.2 Deficient: 5.4 ± 1.0 Insufficient: 5.1 ± 0.9 Sufficient: 4.9 ± 0.7	Caucasian: 5.0 ± 0.8 South Asian: 5.3 ± 0.9	0.002	0.05
Glucose (2 h)	Severely Deficient: 7.6 ± 1.3 Deficient: 7.4 ± 1.2 Insufficient: 7.2 ± 1.7 Sufficient: 7.0 ± 1.5	Caucasian: 7.0 ± 2.2 South Asian: 8.1 ± 2.2	0.02	0.0007

**Table 2:** Comparative analysis of maternal and neonatal parameters by ethnicity. This table presents data on maternal vitamin D (Vit D) levels, glucose tolerance test (GTT) results at fasting (0 min) and after 120 min, body mass index (BMI), and neonatal birth weight in grams for Caucasian and South Asian (S. Asian) populations. Mean values are reported for Vit D, GTT, BMI, and baby weight, while *p*-values indicate statistical significance between groups. Notable differences include higher GTT-120 min values in the South Asian group (8.11 vs. 7.03; *p* = 0.0007) and a lower BMI (28.64 vs. 31.24; *p* = 0.01). Neonatal birth weight was also significantly lower in South Asian neonates (3058 g vs. 3308.5 g; *p* = 0.004). Statistical significance was assessed using appropriate tests, with *p* < 0.05 considered significant.

	Vit D	GTT—0 min	GTT—120 min	BMI (kg/m^2^)	Baby Weight (g)
Caucasian	45.15	5.05	7.03	31.24	3308.5
South Asian	41.17	5.28	8.11	28.64	3058
*p*-value	0.08	0.01	0.0007	0.01	0.004

**Table 3:** Vitamin D levels stratified by season and ethnicity. Vitamin D levels (nmol/L) presented as the mean ± standard deviation (SD) for different groups. Seasonal variation shows significantly lower levels in winter (36.1 ± 12.3 nmol/L). An ethnicity comparison reveals significant differences in vitamin D levels between Caucasian and other groups (*p* = 0.01). A group-wise comparison for seasonal variation indicates strong statistical significance (*p* < 0.001).

Variable	Group/Category (Mean ± SD)	Ethnicity (Mean ± SD)	*p*-Value (Group Comparison)	*p*-Value (Ethnicity Comparison)
Vitamin D (nmol/L)	Winter: 36.1 ± 12.3	Caucasian:	<0.001	0.01
	Summer: 48.7 ± 15.8	Winter: 39.4 ± 13.2 Summer: 51.8 ± 14.9		
		South Asian:		
		Winter: 31.2 ± 11.8 Summer: 43.7 ± 16.5		

## Data Availability

The data presented in this study are available upon request from the corresponding author. The data are not publicly available due to privacy and ethical restrictions.
